# Effect of stress factors associated with postharvest citrus conditions on the viability and biocontrol activity of *Clavispora lusitaniae* strain 146

**DOI:** 10.1371/journal.pone.0239432

**Published:** 2020-09-18

**Authors:** Martina María Pereyra, Mariana Andrea Díaz, Friedhelm Meinhardt, Julián Rafael Dib

**Affiliations:** 1 Planta Piloto de Procesos Industriales Microbiológicos (PROIMI-CONICET), Tucumán, Argentina; 2 Institut für Molekulare Mikrobiologie und Biotechnologie (IMMB), Westfälische Wilhelms Universität Münster, Münster, Germany; 3 Instituto de Microbiología, Facultad de Bioquímica, Química y Farmacia, Universidad Nacional de Tucumán, Tucumán, Argentina; Universita degli Studi di Pisa, ITALY

## Abstract

Only quite recently, we have shown that yeast strains *Clavispora lusitaniae* 146 and *Pichia fermentans* 27 can act as efficient biocontrol agents for combating postharvest fungal diseases in lemons. During postharvest and storage conditions, microorganisms are subject to different stress factors that could affect both their survival and their protective capacity. Understanding the tolerance of yeasts to environmental stress factors could support the future development and commercial application of biological control formulations based on such organisms. Thus, the impact of different stressors on the viability and protection efficiency of *C*. *lusitaniae* strain 146 and *P*. *fermentans* strain 27 was evaluated, and the yeasts were subjected to oxidative stress, thermal treatments, exposure to NaOCl, osmotic stress, and ultraviolet irradiation. *Candida oleophila* strain O served as the reference control. *C*. *lusitaniae* 146 was more resistant to H_2_O_2_ in plate assays; however, in liquid media there was no significant difference to the other strains. Strain 146 was less affected by NaOCl, being able to survive with 300 ppm. *P*. *fermentans* 27 was the strain most heavily affected by osmotic pressure, while strains 146 and strain O showed a similar adaptation. UV-B irradiation severely affected *C*. *oleophila* and *P*. *fermentans*, while *C*. *lusitaniae* was the most resistant. Strains 146 and 27 were similarly tolerant to thermal shocks, compared to the reference strain, which was less viable. In *in vivo* tests, exposure to 10 mM H_2_O_2_, 45°C or 200 ppm NaOCl prior to fruit inoculation, reduced the antagonistic activity against the pathogen *Penicillium digitatum*. However, in no case was the biocontrol efficiency reduced to less than 50%. As *C*. *lusitaniae* 146 demonstrated a great potential to combat *P*. *digitatum* under a wide range of conditions, the organism is a promising candidate as an effective and valuable alternative to toxic fungicides.

## Introduction

Microbes displaying biocontrol activities against fungal pathogens have repeatedly been used for preventing postharvest diseases occurring during the storage and transport of fruits and vegetables [1–5]. Due to their proven safety they shaped up as a powerful alternative to chemical fungicides [6, 7], concomitantly offering the possibility of promoting sustainable agriculture based on organic fruit production [8]. Among the different microbial biocontrol agents, antagonistic yeasts and research on them were prioritized [9] as they regularly devoid of mycotoxins or allergenic spores [10]. The majority of such yeasts are not pathogenic for humans [11] and they can grow in environments with low water activity as well as limited nutrient availability and low oxygen levels [12]. *Candida oleophila* is considered a biocontrol model for fighting postharvest fruit diseases and, consequently, commercial products have been released to the market and are still available [13–15]. Besides *C*. *oleophila*-containing products, which have proven to have certain efficiency in citrus protection, so-called killer yeasts, highly efficient in the specific protection of lemons against fungal postharvest infections, have been isolated only quite recently. *Clavispora lusitaniae* strain 146 [16, 17] and *Pichia fermentans* strain 27 [18] isolated from lemon fruits arose as biocontrol agents for combating the green mold *Penicillium digitatum*, the most relevant fungus causing postharvest diseases in lemon.

Contrary to chemical agents, the action of yeasts as biocontrol agents largely depends on the prevailing environmental conditions and the organisms´ response to ecological parameters (temperature, salt stress, oxidative stress, UV-B irradiation) [19]. Solar radiation, for instance, impacts the survival of the antagonist [20]. The lemons industry routinely keeps postharvest and storage conditions as stable as possible [21]; however, inherent to the system, fruits are exposed to different factors that influence the performance of the biocontrol agent [22]. During packaging, chemical disinfection [23] and drying processes [2] can significantly impact the yeast cells viability. Moreover, oxidative stress triggered by reactive oxygen species, such as hydrogen peroxide (H_2_O_2_) emerging in wounds of the fruit, can negatively affect the protective effects [24]. Hence, knowledge of the conditions for the optimal growth and development is crucial to guarantee a level of protection that meets the requirements of the industry [8]. Since the ability of the killer yeasts to resist conditions associated with packaging processes that affect their preventive capacity is unknown, the purpose of this work is to study the response of biological control strains *C*. *lusitaniae* 146 and *P*. *fermentans* 27 to stress conditions routinely associated with postharvest and packaging of the lemon fruits in comparison to the reference *C*. *oleophila* strain O.

## Materials and methods

### Microorganisms

The organisms used in this study were *Clavispora lusitaniae* strain 146 (NCBI accession number KY442860) and *Pichia fermentans* strain 27 (KY442834), previously isolated from citrus fruits and selected due to their capability to function in biological control of the green mold disease of lemons [18]. *Candida oleophila* strain O [14] was used as the reference control. The organisms were cultivated in liquid Yeast Extract Peptone Dextrose (YEPD) medium (pH 4.5) and stored on YEPD agar plates at 8°C.

As the biocontrol target organism *Penicillium digitatum* from the citrus fungi collection of the Phytopathology Laboratory of San Miguel S.A citrus company was selected due to its postharvest aggressiveness for lemon fruits. Spore suspensions were obtained after inoculation of lemon fruits with *P*. *digitatum*. When fruits were completely covered by the fungus (usually after 5 days at 25°C) spores were collected in standard saline solution containing 0.1% Tween 80.

### Yeasts survival evaluation under stress conditions

#### Sensitivity of yeast cells to oxidative stress induced by hydrogen peroxide (H_2_O_2_)

The sensitivity assay of *C*. *lusitaniae* 146, *P*. *fermentans* 27 and *C*. *oleophila* strain O to oxidative stress followed the protocol established by Spencer et al. [25], with modifications. Cultures were grown in YEPD liquid medium at 25°C for 24 h and adjusted to a final concentration of 10^8^ cells/mL using a Neubauer cell counting chamber. For evaluating resistance levels, 5 μL of ten-fold serial dilutions of each of the strains were spotted on YEPD agar containing 1 mM, 2.5 mM, 5 mM, 10 mM, 12.5 mM and 15 mM of H_2_O_2_. For control purposes, cells were spotted on YEPD agar without H_2_O_2_ and—as for the H_2_O_2_ containing samples—incubated for 48 h at 25°C prior to growth determination.

Monitoring viability: 20 mL suspensions (10^8^ cells/mL) were exposed to different H_2_O_2_-concentrations (5 mM, 10 mM, 20 mM, 100 and 200 mM) and cultivated in an orbital flat shaker (Bioamerican Science, BS 875) for 15 min at 120 rpm. Cells were pelleted and washed twice with saline solution before being serially diluted. 100 μL of yeast suspensions (10^8^ cells/mL) were plated on YEPD agar. Viability was monitored by counting colonies occurring after 48 h of incubation at 25°C. Both, the spot assays and the viability tests were done in triplicate.

#### Survival of yeast cells after sodium hypochlorite (NaOCl) treatment and exposure to sodium chloride (NaCl)

Yeasts sensitivity to sodium hypochlorite was assessed as described in Kwolek-Mirek et al. [26]. As for the H_2_O_2_ assays cells were grown in YEPD medium for 24 h and adjusted to 10^8^ cells/mL. Cultures were ten-fold serially diluted and 5 μL of each dilution were spotted on solid YEPD medium containing 0 ppm, 50 ppm, 100 ppm, 150 ppm, 200 ppm, 300 ppm and 400 ppm of NaOCl. Subsequently, plates were incubated at 25°C for 48 h and growth was monitored.

Monitoring viability: 20 mL of liquid cultures (10^8^ cells/mL) containing 100 ppm, 200 ppm, 300 ppm and 400 ppm of NaOCl were shaken (Bioamerican Science, BS 875) at 120 rpm for 15 min. Subsequently, cultures were washed twice with saline solution, serially diluted and 100 μL of yeast suspensions were plated on YEPD agar. Petri dishes were incubated under the same conditions as described above. The number of CFUs per milliliter (CFU/mL) was determined after 48 h at 25°C.

Assessment of the influence of saline stress on the yeast strains: cells were exposed at increasing time-intervals to 6 M NaCl essentially as described in Wang et al. [23]. 10 mL cell suspensions (10^8^ cells/mL) were pipetted into a 50 mL centrifuge tube and exposed to 6 M NaCl for 30, 60, 120, 180 and 240 min at 25°C on a rotary shaker (Bioamerican Science, BS 875) at 120 rpm. At the above timely intervals, 100 μL of ten-fold serial dilutions were spread on solid YEPD and incubated for 48 h prior to determining the CFU/mL. All assays were carried out in triplicate.

#### Effect of UV-B irradiation on the viability of the biocontrol yeast strains

For determining the effect of UV-B irradiation on the yeast strains used in this study, spot assays were performed. As for above assays ten-fold serial dilutions of each strain culture (10^8^ cells/mL) were spotted on YEPD medium and after inoculation immediately exposed to UV-B irradiation (using a Vilbert Lourmat VL-4 lamp with maximum intensity at 312 nm) essentially as described in Portero et al. [27]. Petri dishes were covered with cellulose acetate film to get rid of the UV-C part. Inoculated plates were exposed to UV-B irradiation at 2.98 W/m^2^ for 0 h, 0.5 h (5.36 Kj/m^2^), 1 h (10.73 Kj/m^2^), 1.5 h (16.09 Kj/m^2^), 2 h (21.46 Kj/m^2^), 2.5 h (26.82 Kj/m^2^) and 3 h (32.18 Kj/m^2^). Subsequently, strains were immediately kept in the dark for 48 h at 25°C to prevent photoreactivation.

The relative growth inhibition was estimated as follows: four plus signs (++++) when the yeasts´ growth was the same as the control, three plus signs (+++) when was slightly less than the control, two plus signs (++) when was partial inhibition, one plus sign (+) when a strong inhibition was; or with a negative sign (-) when no growth was observed.

#### Assaying thermotolerance after heat shock

Thermal treatment was done according to Liu et al. [28], with some modifications: yeast cell suspensions (10^8^ cells/mL) were subjected to 45°C and 50°C for 3 min, 5 min and 10 min. Immediately after heat treatment, cells were cooled on ice and serially diluted for proper determination of the CFU/mL on YEPD agar. The control consisted of yeast cell suspensions that were not exposed to any heat treatment (0 min). All plates were incubated at 25°C for 48 h followed by colony counting.

### Capability test for biocontrol of stressed yeast strains with *P*. *digitatum* as the target

Biocontrol activities of *C*. *lusitaniae* 146, *P*. *fermentans* 27 and *C*. *oleophila* strain O was performed as described by Perez et al. [18]. Lemon fruits were disinfected with 70% ethanol solution and were wounded in the equatorial zone by using an awl (one wound per lemon). 20 μL-suspensions (10^8^ cells/mL) of non-treated or treated yeast cells (with 10 mM of H_2_O_2_ for 15 min, 200 ppm of NaOCl for 15 min or heated 5 min at 45°C) was applied to each wounded site. After 24 h of incubation at 25°C, each of the fruits was inoculated at the wounded site with 20 μL of a spore suspension of *P*. *digitatum* (1x10^6^ spores/mL). The fruits were subsequently kept stored in closed plastics bags under high humidity (95%) at 25°C for 5 days. Three replicates with ten lemon fruit for each treatment were performed. Ten additional lemons were taken as the control; they were wounded and inoculated solely using the spore suspension of the phytopathogenic *P*. *digitatum*.

### Statistical analysis

All experiments were repeated at least three times. The data were analyzed by ANOVA, and the mean values were compared with Tukey’s test at the 5% significance level. The InfoStat/L software [29] was used for the statistical analysis.

## Results

### Sensitivity to hydrogen peroxide-induced oxidative stress

**Fig 1:**
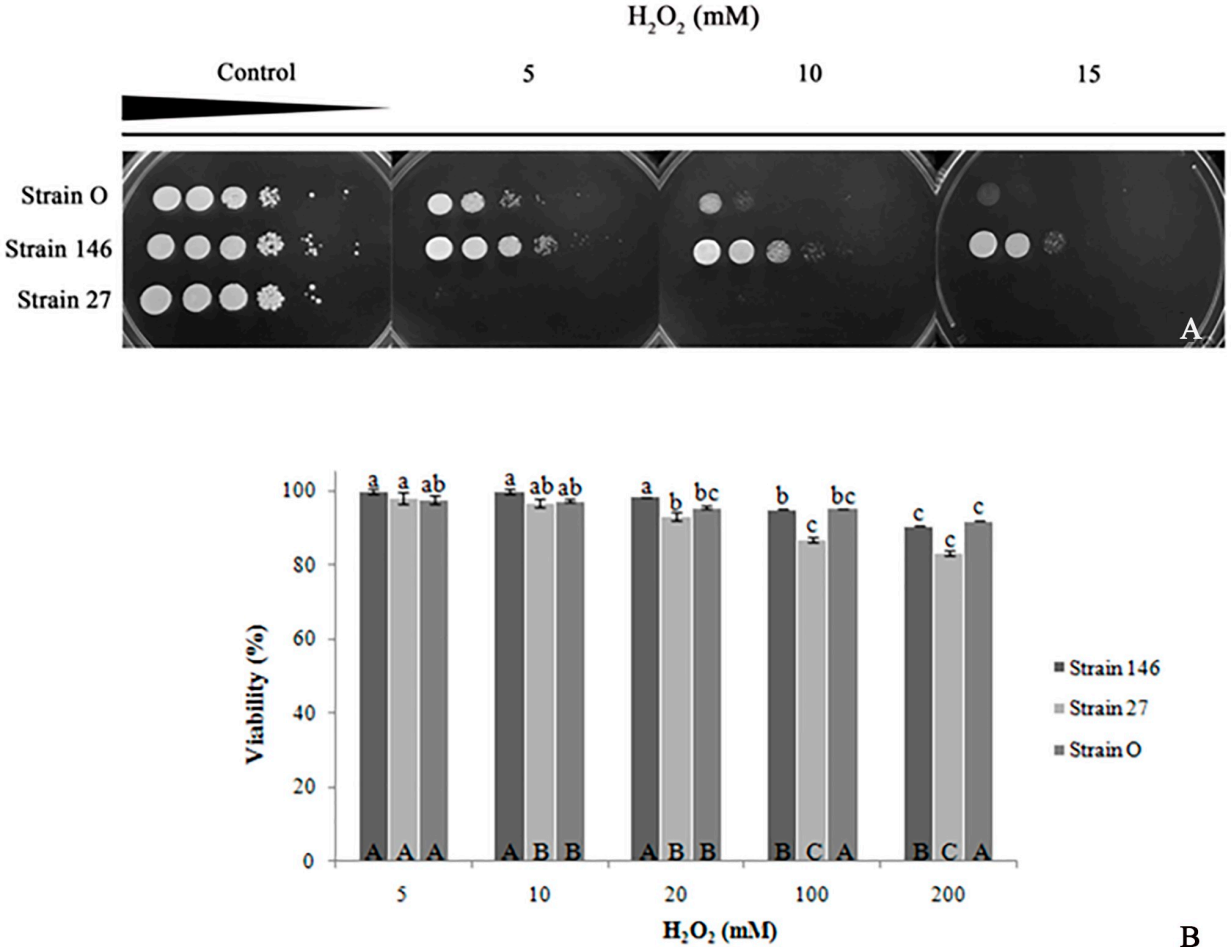
Impact of oxidative stress on biocontrol yeasts. (A) Liquid YEPD-cultures of *C*. *lusitaniae* 146, *P*. *fermentans* 27 and *C*. *oleophila* strain O were ten-fold serially diluted and spotted onto YEPD agar containing the above specified H_2_O_2_ concentrations. Pictures were taken after 48 h at 25°C. (B) Viability of yeast cells referred in relation to the untreated control. Yeasts were exposed to the below specified H_2_O_2_ concentrations for 15 min. For details see Material and Methods 2.2.1. Mean values marked with identical letters are—according to the Tukey test (p <0.05)—not significantly different. Lowercase letters refer to the comparison between the maltreated strains and the respective control. Uppercase letters represent the comparison between yeasts at a certain condition.

When the biocontrol strains were exposed to increasing H_2_O_2_ concentrations in solid medium it turned out that, in each case, viability was negatively affected by the oxidative agent (Fig 1A). While *P*. *fermentans* 27 was already completely inhibited by 5 mM H_2_O_2_, *C*. *oleophila* strain O survived up to 10 mM. *C*. *lusitaniae* 146 displayed the most prominent resistance to the hydrogen peroxide-induced oxidative stress as it survived the highest concentration tested (15 mM H_2_O_2_). All of the strains were able to survive 200 mM H_2_O_2_ when exposed for 15 min (Fig 1B). At 20 mM H_2_O_2_, the viability of *C*. *oleophila* strain O and *P*. *fermentans* 27 decreased compared to *C*. *lusitaniae* 146; a significant decrease occurred already in 100 mM H_2_O_2_. At 200 mM, viability of strain 146 (90.44%) and strain O (91.94%) was significantly greater than that of strain 27 (83.12%).

### Effect of sodium hypochlorite and 6 M sodium chloride on yeasts viability

**Fig 2:**
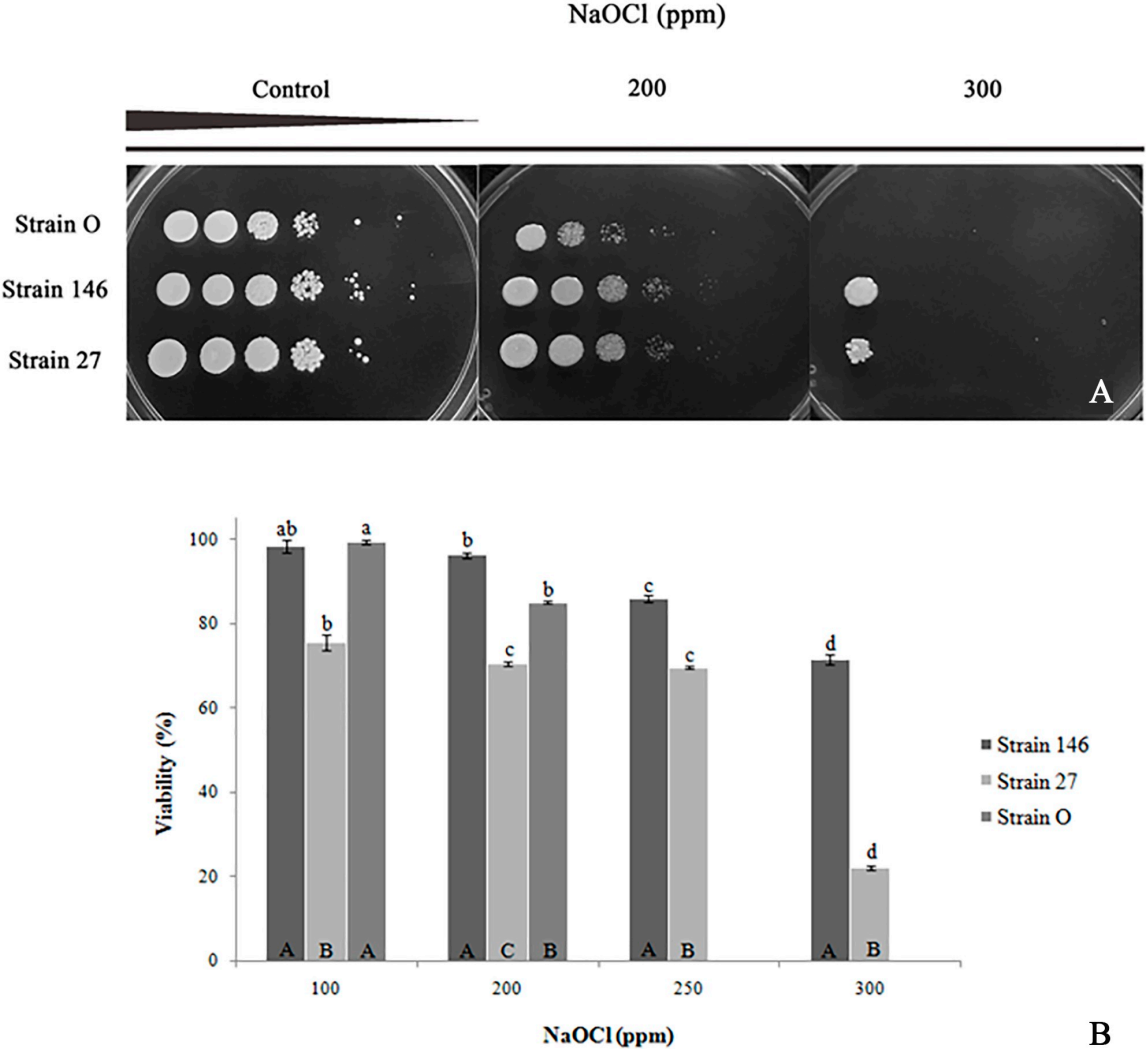
Effect of different NaOCl concentrations on the survival of *C*. *lusitaniae* 146, *P*. *fermentans* 27, and *C*. *oleophila* strain O. (A) The organisms were grown on YEPD liquid medium, ten-fold serially diluted and spotted onto YEPD agar plates containing above specified concentrations of the disinfectant. Growth monitoring as in Fig 1. (B) For viability assays, strains were exposed for 15 min to 100 ppm, 200 ppm, 250 ppm, 300 ppm and 400 ppm NaOCl and survival was expressed in relation to the respective untreated control. Statistic evaluation as for Fig 1. Lowercase letters again correspond to the comparison between a strain and the respective control. Uppercase letters represent comparisons between yeasts at the specific conditions.

When NaOCl was added to the solid medium, growth of *C*. *lusitaniae* 146 and *P*. *fermentans* 27 was seen up to 300 ppm (Fig 2A), both of the organisms turned out to be more tolerant to NaOCl than *C*. *oleophila* strain O, which survived only up to 200 ppm. Addition of NaOCl to liquid medium generated comparable results (Fig 2B). *C*. *oleophila* strain O tolerated up to 200 ppm of NaOCl with a viability of 84.96%, which is significantly lower than for *C*. *lusitaniae* 146 but greater than for *P*. *fermentans* 27 (96.19% and 70.49%, respectively); however, it failed to grow at higher concentrations. Resistance was observed for *C*. *lusitaniae* 146 even at 300 ppm, at least displaying a viability of 71.41%, unlike *P*. *fermentans* 27 that only reached 22.16% under the same conditions. 400 ppm NaOCl sufficed to inhibit growth at all.

**Fig 3:**
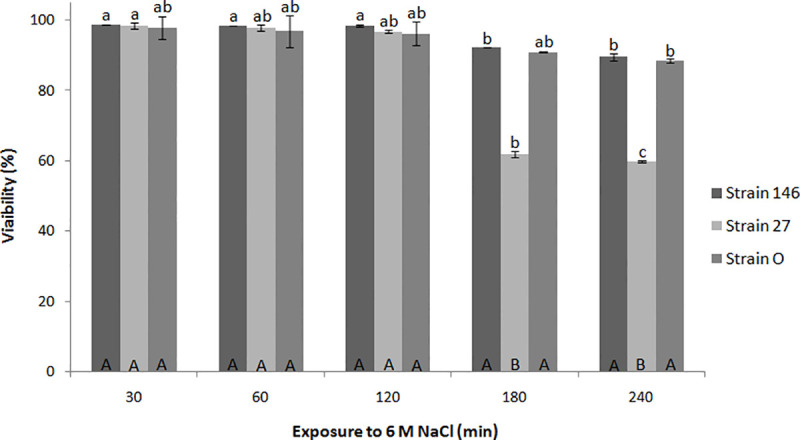
Survival of yeast strains after temporary exposure to 6 M NaCl solution. Statistical evaluation as for Fig 1. Lowercase letters again correspond to the comparison between a strain and the respective control. Uppercase letters represent comparisons between yeasts at the specific conditions.

With respect to the survival after exposure to (lethal) 6 M NaCl, it turned out that—though the strains were able to tolerate the longest exposure time tested (240 min)—*C*. *oleophila* strain O and *C*. *lusitaniae* 146 performed significantly better than *P*. *fermentans* 27 (Fig 3). Viability of strain 146 decreased by 10.40%, similar to strain O (11.5%); strain 27 showed the lowest tolerance to NaCl as its viability decreased by 40% after 240 min in the concentrated saline solution.

### Effect of UV-B irradiation on the *in vitro* viability of yeasts

**Fig 4:**
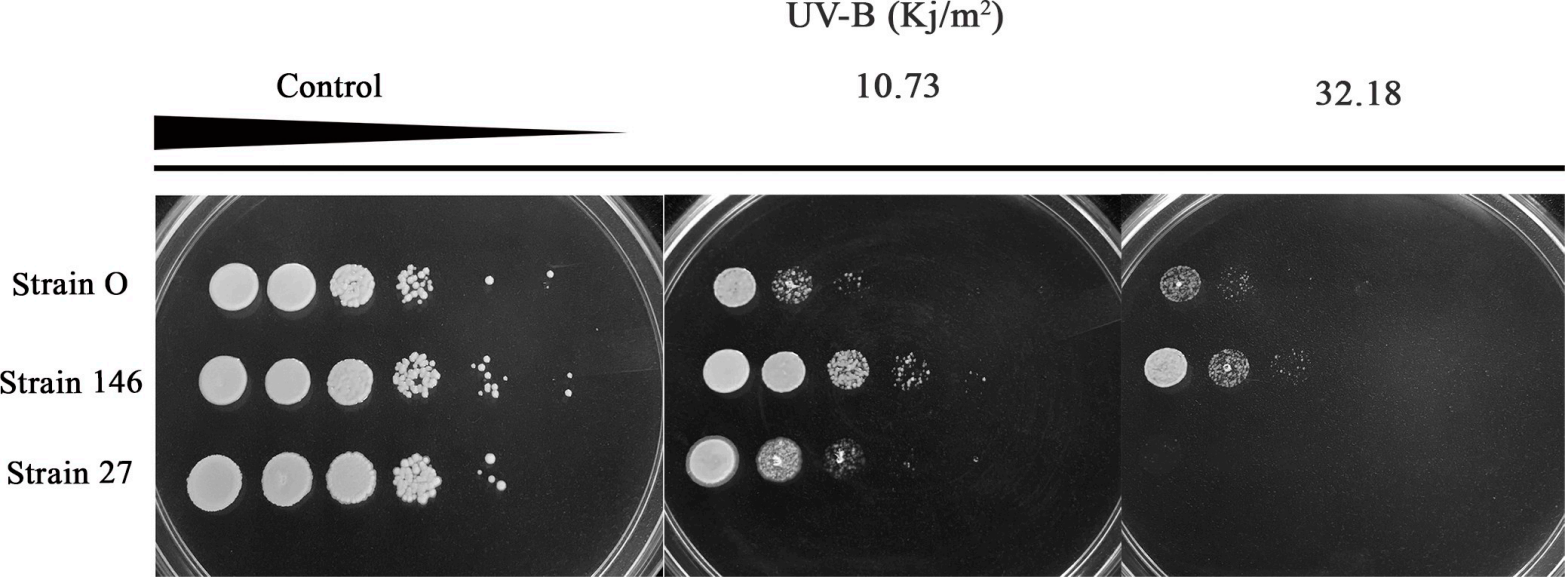
Growth assessment after UV-B irradiation of yeasts cells exposed to different dosages. Cells were ten-fold serially diluted, and 5 μL of each dilution was spotted onto YEPD agar. Subsequently, the plates were irradiated with UV-B at the indicated doses: 5.36 Kj/m^2^ (0.5 h), 10.73 Kj/m^2^ (1 h), 16.09 Kj/m^2^ (1.5 h), 21.46 Kj/m^2^ (2 h), 26.82 Kj/m^2^ (2.5 h) and 32.18 Kj/m^2^ (3 h). The figure exemplarily shows the control, without any exposure to UV-B, and those that been exposed for 1 h (10.73 Kj/m^2^) and 3 h (32.18 Kj/m^2^), respectively.

For testing their tolerance to different intensities of UV-B irradiation the yeast strains were exposed to increasing energy doses. Results presented in Fig 4 and Table 1 show that—as expected—UV-B negatively influences survival in each case. Exposure to 10.73 Kj/m^2^ already severely hit *C*. *oleophila* strain O and *P*. *fermentans* 27 while *C*. *lusitaniae* 146 performed much better. Such effect is seen more pronounced at 32.18 Kj/m^2^ in Fig 4. Again *C*. *lusitaniae* 146 withstands the treatment much better than both of the other strains thereby confirming the results obtained at 10.73 Kj/m^2^. Fig 4 shows the outcome of assays in which 5 μL of ten-fold serial dilutions of cell suspensions containing 10^8^ cells/mL were immediately irradiated after spotting. Table 1 summarizes results of more experiments performed with increasing UV-B doses. As can be seen from Fig 4 and Table 1, *C*. *lusitaniae* 146 scored best when exposed to UV-B.

**Table 1:** Survival of yeast strains exposed to UV-B irradiation.

UV-B doses (Kj/m^2^)	Strain O	Strain 146	Strain 27
**Control**	++++	++++	++++
**5.36**	+++	+++	+++
**10.73**	++	+++	++
**16.09**	++	+++	++
**21.46**	++	+++	+
**26.82**	+	++	-
**32.18**	+	++	-

The number of + signs refer to growth assessments: ++++, growth of the control (see also Fig 4 on the left side); +++ slightly lesser growth than for the control (four to five spots displaying growth as for *C*. *lusitaniae* in Fig 4, middle part); ++ more severe inhibition (growth observed in two or three spots); + strong inhibition (only one spot grown);—no growth at all.

### Thermotolerance of the yeast strains

**Fig 5:**
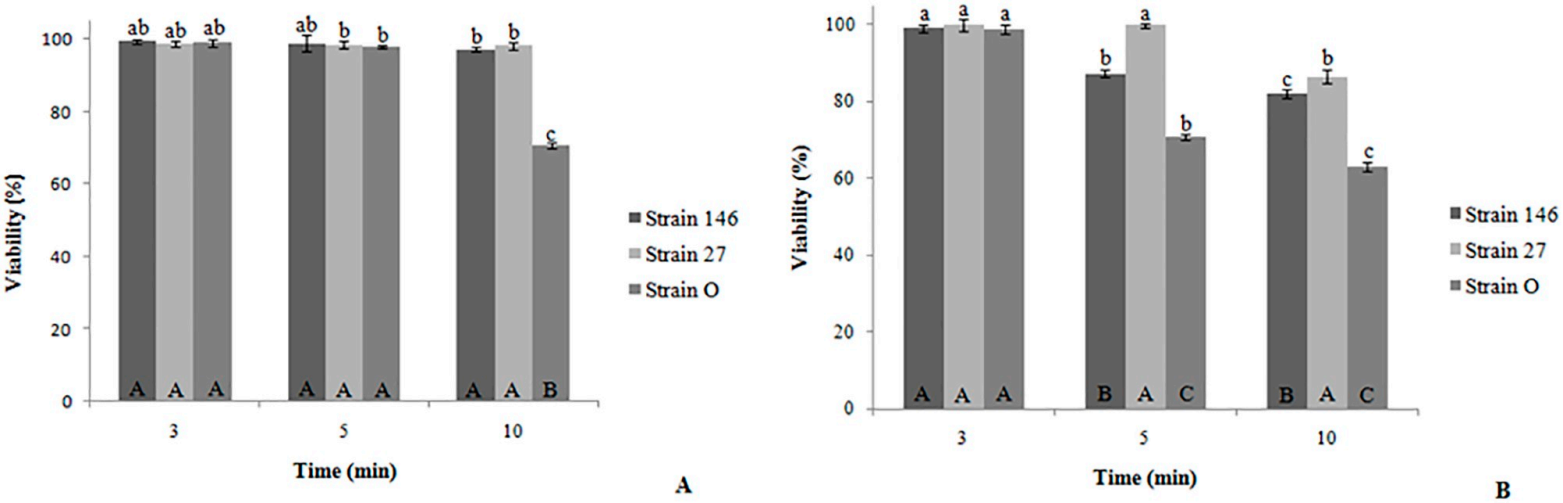
Effect of heat shock treatment on the viability of yeasts. After exposure to 45°C (A) and 50°C (B) for 3, 5 and 10 min, cells were ten-fold serially diluted and plated on YEPD agar, followed by incubation at 25°C for 48 h. Mean values marked with identical letters are—according to the Tukey test (p <0.05)—not significantly different. Lowercase letters correspond to the comparison between each stressed yeast and its control. Uppercase letters represent the comparison between yeasts at a certain condition.

For mimicking conditions during drying and waxing processes in lemon packinghouses, cells were exposed to thermal treatments for increasing periods of time. *C*. *lusitaniae* 146 and *P*. *fermentans* 27 withstood the 45 and 50°C treatments better than *C*. *oleophila* strain O (Fig 5A and 5B). As shown in Fig 5A, *C*. *oleophila* strain O and *P*. *fermentans* 27 resisted the heat up to 3 min without significant changes at 45°C, while *C*. *lusitaniae* 146 coped with such condition up to 5 min. After 10 min, strain 146 and strain 27 still displayed 97.11% and 98.12% viability, respectively, which is significantly higher than for strain O (70.67%). When the yeasts were subjected to 50°C (Fig 5B), the decrease of viability was more pronounced. After 5 min, a significant impact was seen for the strains 146 and O, whereas for *P*. *fermentans* 27 such decrease occurred only after 10 min. Thus, strain 27 most efficiently withstood the high temperatures, still displaying 98.12% and 86.54% survival rates at 45 and 50°C, respectively.

### Biocontrol assay of stressed yeasts against *P*. *digitatum*

**Fig 6:**
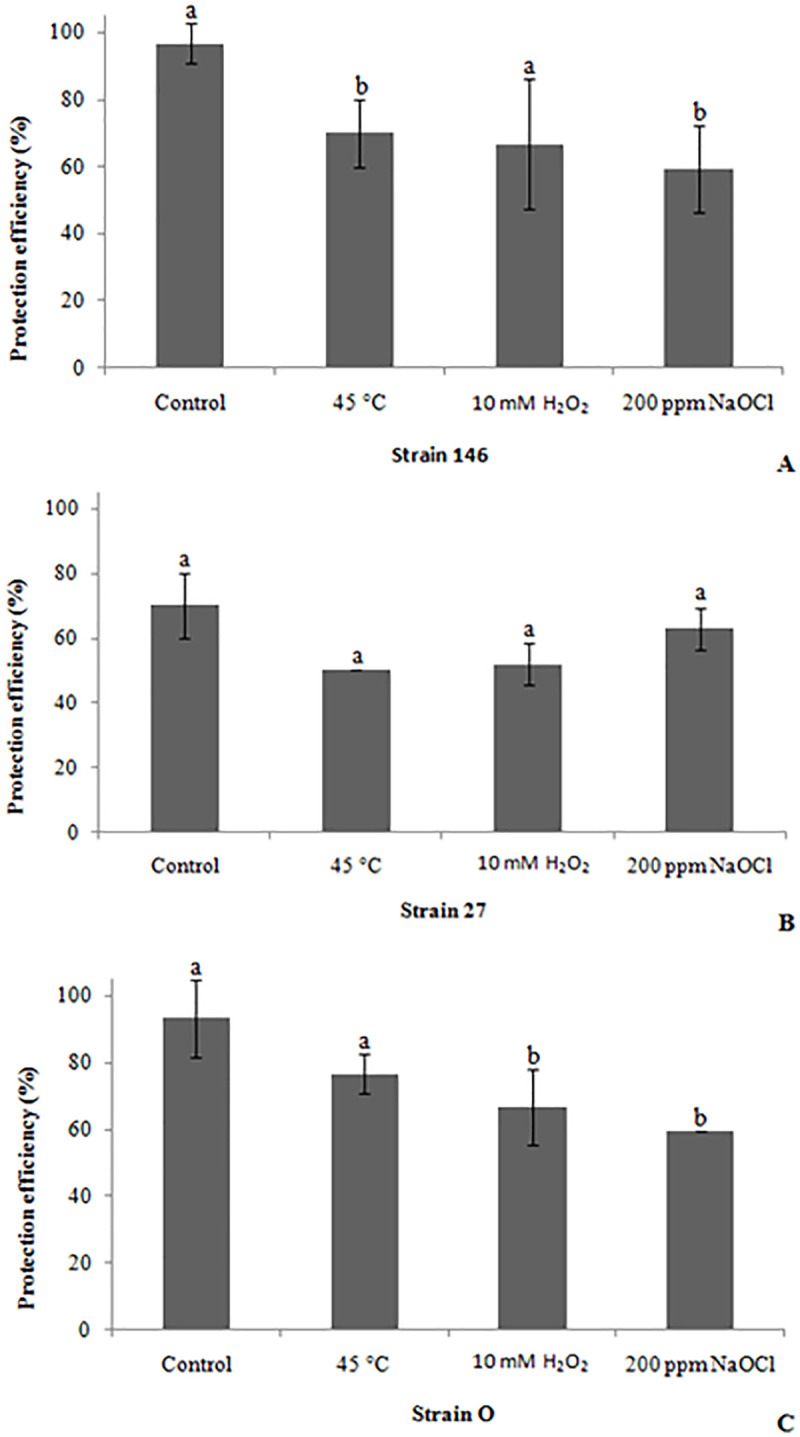
Biocontrol efficiency of stressed yeasts against *P*. *digitatum*. *C*. *lusitaniae* 146 (A), *P*. *fermentans* 27 (B) and *C*. *oleophila* strain O (C) were exposed to different stress factors (45°C for 5 min, 10 mM H_2_O_2_ for 15 min and 200 ppm NaOCl for 15 min) before evaluating its efficiency in controlling the pathogen. Mean values marked with identical letters are—according to the Tukey test (p <0.05)—not significantly different.

All the tested yeasts were exposed to selected stress conditions prior to evaluating *in vivo* their protecting capabilities against the phytopathogenic *P*. *digitatum* in lemons. The conditions applied were: exposure to 45°C for 5 min, exposure to 10 mM H_2_O_2_ for 15 min, and exposure to 200 ppm NaOCl for 15 min. All of the stressors affected the protecting efficiency. Regarding *C*. *lusitaniae* 146, exposure to NaOCl was the factor that most influenced the yeast biocontrol efficiency, since the obtained 59.26% was significantly lower than for cells without such treatment (Fig 6A). Both, thermal shock and H_2_O_2_ decreased the *in vivo* antagonistic activity of strain 146, but only the former significantly affected the viability with respect to the control. For *P*. *fermentans* 27 (efficiency in wound protection was 70% when not subjected to any stress) it turned out that—though the value is markedly lower than for the untreated strain 146 (96.67%) -neither the temperature, nor the oxidative stress induced by H_2_O_2_, nor exposure to NaOCl significantly affected the protection efficiency (Fig 6B). Finally, results presented in Fig 6C prove that the protection conferred by *C*. *oleophila* strain O was 66.67% and 59.26% when exposed to H_2_O_2_ and 200 ppm NaOCl, respectively, showing significant differences to the control. However, when the latter yeast was stressed by temperature, it still reached a protection efficiency of 76.67%, which does not differ significantly from that obtained in the control.

## Discussion

As we have previously shown, *P*. *fermentans* 27 and *C*. *lusitaniae* 146 can efficiently prevent postharvest fungal infections in lemons. Particularly the latter—due to its higher and consistent antagonistic activity—has been studied in some more detail. *C*. *lusitaniae* 146 colonizes wounded fruit, inhibits *P*. *digitatum* spore germination and it tolerates the waxes used in the citrus industry. Moreover, the strain depicts preventing efficacy even against a fungicide-resistant *P*. *digitatum*; even heat-inactivated cells of such yeast retain a certain level of control against fruit decays [16, 17].

Here—by comparing it with the reference *C*. *oleophila* strain O—we analyzed the response of such antagonistic yeasts (*C*. *lusitaniae* 146 and *P*. *fermentans* 27) to various modeled stress factors associated with pre- and postharvest conditions and packaging of lemon fruits. *C*. *oleophila* strain O, the reference, was originally isolated from apples and first studies demonstrated its biocontrol activity against *Penicillium expansum* and *Botrytis cinerea* in apples and pears [14]. Its antagonistic capacity against citrus postharvest pathogens, such as *P*. *digitatum* and *Penicillium italicum*, was evaluated by Droby et al. [13]. Two commercial *C*. *oleophila*-based products with an extended spectrum of action for protecting different fruits are currently available. The species is considered as the reference in biological control studies as its response to various stressors, such as water stress and low relative humidity has been studied [30]; more recently, Lahlali et al. [21] evaluated its response to UV-B irradiation.

Biological control agents can be significantly affected by several environmental factors [31]; oxidative stress being, undoubtedly, one of the most important factors as the fitness and the suitability of an organism for postharvest biocontrol depends on its capacity to resist oxidative stress [19]. In the present report we show that effects of the oxidant on growth are more severe in solid than in liquid medium. Viability assays in liquid medium revealed that survival of *C*. *lusitaniae* 146, *P*. *fermentans* 27 and *C*. *oleophila* strain O treated with 200 mM H_2_O_2_ for 15 min were 90.44%, 83.12% and 91.94%, respectively. Findings reported by Liu et al. [28, 32, 33] strongly support our results; studying the tolerance of three antagonistic yeasts to oxidative stress they reported that the yeast *Metschnikowia fructicola* was most tolerant, displaying survival rates up to 88% after 20 min in 200 mM H_2_O_2_ [28]; the yeasts *C*. *oleophila* strain I-182 [32] and *Cystofilobasidium infirmominiatum* [33] reached only 28% at 100 mM H_2_O_2_ and 23% in 20 mM H_2_O respectively, which are—despite of having used the same methodology in all cases—rather low values compared to those of the yeast strains studied here. Spencer et al. [25] examined the tolerance of yeasts to oxidative stress induced by H_2_O_2_ and showed that the yeasts *Pichia guilliermondii*, *Candida shehatae*, *Candida succiphila* and *Scheffersomyces stipitis*, when exposed to concentrations between 1 and 7.5 mM of H_2_O_2_ for 15 min, did not display a viability greater than 20% in any case, which is indicative of the high sensitivity of those yeasts to the oxidative stress, particularly when compared to the strains examined in this work.

For fighting bacteria and fungi, NaOCl at a concentration of 200 ppm is routinely added to the wash water in lemon packinghouses at the beginning of the packing process. Indeed, the compound is active against *Staphylococcus aureus*, *Enterococcus faecalis*, *Pseudomonas aeruginosa*, *Bacillus subtilis*, *Listeria monocytogenes*, *Salmonella enteric* [34], *Candida albicans*, *Alternaria alternate* [35–38] and *Saccharomyces cerevisiae* [39, 40]. The availability of biocontrol yeast strains able to tolerate such NaOCl concentrations is, thus, considered an important quality. As shown in this work, strains *C*. *lusitaniae* 146 and *P*. *fermentans* 27 survive 300 ppm NaOCl, the maximum concentrations tested. Strain 27 retained a viability of 22.16% while strain 146 lost only 30% of viability. For the reference strain *C*. *oleophila*, the NaOCl concentration used in packaging (200 ppm), resulted in a viability of 85%, but at higher concentrations, it hardly survived. Matching results were obtained in solid medium. Analogously, Kwolek-Mirek et al. [26] showed that NaOCl causes growth inhibition and loss of viability as well as metabolic activity in both wild-type yeast and antioxidant deficient mutants. However, unlike our findings, they reported the oxidant to be more effective at lower concentrations in liquid compared to solid medium, a finding that probably reflecting chemical reactions of the NaOCl with components of the solid medium.

When Wang et al. [23] performed a mild salt stress pretreatment of the strain *C*. *oleophila* I-182 to check whether such treatment increases the tolerance to the subsequent exposure to a lethal saline stress (6 M), they determined viability percentages for *C*. *oleophila* I-182 not adapted to stress reaching 52.2%, 41.2% and 34.6% for 30, 60 and 90 min, respectively, whereas cells with the saline pretreatment obtained values of 79.7%, 75.1% and 52.7%. In our study with *C*. *oleophila* strain O and for *C*. *lusitaniae* 146 after 240 min in 6 M NaCl, without a precedent saline adaptation treatment, we obtained viability values of 88.48% and 89.60%, respectively. Moreover, even for the maximum salt exposure time, the viability of *P*. *fermentans* 27 (59.91%) was greater than for *C*. *oleophila* I-182 not-stress-adapted at all, but less than that of the adapted cells after the first two exposure times. Thus, the killer yeasts investigated in this study are highly and more resistant to saline stress than the other known strains.

UV-B irradiation (280–320 nm), responsible for direct and indirect DNA damages, is among the most significant factors limiting survival of biocontrol agents under field conditions [2, 41, 42]. In the work carried out here, *in vitro* exposure to UV-B for 2 h or more (21.46 Kj/m^2^) resulted in the complete loss of viability of *P*. *fermentans* 27, while *C*. *lusitaniae* 146 after 3 h (32.18 Kj/m^2^) showed a greater tolerance to the irradiation than *C*. *oleophila* strain O. Results obtained for strain O are consistent with study reported by Lahlali et al. [21], which likewise mentions the total loss of viability after 3 h (2.79 Kj/m^2^) of UV-B irradiation despite the fact that the difference in the intensity of irradiation is significant, being notably greater in our study. The effect of UV-B irradiation on *Pichia anomala* strain K was evaluated *in vitro* by Lahlali et al. [20]. Achieving a lethal dose of 90% needed an *in vitro* radiation at 1.6 Kj/m^2^, a finding that allows for strengthening the versatility of the yeasts studied here because for both, strain *C*. *lusitaniae* 146 and strain *C*. *oleophila* O, a markedly higher dose (32.18 Kj/m^2^) was required to obtain such low viability. Knowledge of the response of biocontrol agents to various environmental stresses is crucial for identifying antagonistic activity and the factors limiting survival of a biocontrol agent survival when applied as preharvest treatments [41].

As already mentioned, fruits are exposed to short-time thermal shocks in lemon packinghouses ranging from 45 to 50°C, primarily during drying and waxing. When our strains were thermally stressed, viability of all of the tested yeasts decreased as consequence to the rising temperatures. Nevertheless, they maintained high viability values even after 10 min at 50°C. Survival of *C*. *lusitaniae* 146, *P*. *fermentans* 27 and *C*. *oleophila* strain O were 82.18%, 86.54%, and 62.59%, respectively. Our findings are in line with reports on *C*. *oleophila* strain I-182 [33]; however, 30 min at 41°C depressed its survival to 22%. Viability of *M*. *fructicola* was 87% at 43°C [28] which clearly matches our results for *C*. *lusitaniae* 146 and *P*. *fermentans* 27. The temperature tolerance of two other biocontrol yeasts, *Debaryomyces hansenii* and *Pichia membranaefaciens* [43] was rather low as survival of *D*. *hansenii* dropped to less than 10% after 20 min at 40°C and viability of *P*. *membranaefaciens* reached only approximately 40% after 40 min at 50°C. An even more pronounced loss of viability was obtained for *Pichia guilliermondii* [44] as survival decreased to 4% when cells were heated for 15 min at 45°C. It is noteworthy that, as for the oxidative stress, yeasts differ considerably in their ability to withstand high temperatures, however, *C*. *lusitaniae* 146 and *P*. *fermentans* 27 perform rather robust.

In the biocontrol experiments using wounded lemon fruit, strain 146 and strain O provided higher levels of protection (96.67 and 93.33%, respectively) against *P*. *digitatum* than *P*. *fermentans* 27, the latter reached at least 70%. When yeasts were exposed to the different stressors, in some cases significant differences were observed compared to the respective control; in any case the efficiency in wound protection was less than 50%. More specifically, when cells were subjected to thermal stress (45°C, 5 min), the efficiency of *C*. *lusitaniae* 146, *P*. *fermentans* 27 and *C*. *oleophila* strain O reached 70%, 50%, and 76.67%, respectively. Under similar conditions (45°C, 20 min), Liu et al. [28] investigated the biocontrol activity of yeast *M*. *fructicola* against the phytopathogen *P*. *expansum* in apples determining a disease incidence of 43.3% (or a 56.7% protection efficiency). Such observation is consistent with the one for strain 27 and also reveals the higher temperature tolerance of strain 146 and strain O compared to *M*. *fructicola*. Moreover, we found that, unlike *C*. *oleophila* strain O, both *C*. *lusitaniae* 146 and *P*. *fermentans* 27 exposed to 10 mM H_2_O_2_ showed protection efficiency against *P*. *digitatum* without significant differences to the non-exposed yeasts. Thus, the two strains responded as was suggested by Castoria et al. [24], which stated that oxidative stress resistance is necessary for the yeast to remain viable and maintain biocontrol efficacy in the wounded fruit. Liu et al. [33] studied the effect of a pretreatment with 5 mM H_2_O_2_ for 30 min on the biocontrol activity of the yeast *C*. *oleophila* I-182 and found that the stress-adapted yeast displayed a greater level of efficacy than non-stress-adapted cells in controlling *P*. *expansum* and *B*. *cinerea*. Hypochlorite is an oxychlor compound attacking by oxidation many biological molecules, including proteins, fatty acyl chains, carbohydrates and nucleic acids [26, 37, 45]. The chlorine compound is often used as a disinfectant during the first steps in the citrus packing process and, consequently, it is important to investigate the response of potential biocontrol yeasts to such agent. The three strains exposed to NaOCl exhibited a decrease in the biocontrol of *P*. *digitatum* but, in the case of *P*. *fermentans* 27, there were no significant differences whereas their protection capabilities of *C*. *lusitaniae* 146 and *C*. *oleophila* strain O were notably lowered, reaching both 59.26%.

In this work, the resistance to stress conditions of the killer yeasts *C*. *lusitaniae* 146 and *P*. *fermentans* 27, previously known for their efficiency to prevent postharvest fungal diseases in lemons, was determined and compared to the *C*. *oleophila* strain O. The latter is considered as a reference strain in biocontrol assays and is the main component of well-known commercial formulations used for the biological control of postharvest fungal diseases.

*C*. *lusitaniae* 146 showed an outstanding behavior over the others strains, and by displaying an excellent ability to survive saline or oxidative stress, to resist disinfectants and to maintain viability when exposed to high levels of UV-B irradiation or against thermal shocks, it became a promising candidate for use as a biocontrol agent. Furthermore, it also maintained its capacity to control green mold in lemons even after the previous exposure to diverse stressors.

The capacity of *C*. *lusitaniae* 146 to withstand stress factors associated with preharvest or postharvest conditions, including packing processes, storage, transit or export, makes it a suitable candidate for generating a commercial formulation to combat postharvest fungal infections in lemons or possibly in other citrus fruits as well.
